# Collaborative health education for Somali Bantu refugee women in Kansas City

**DOI:** 10.1186/s13104-019-4649-6

**Published:** 2019-09-23

**Authors:** Ellyn R. Mulcahy, Carla Buchheit, Elyse Max, Suzanne R. Hawley, Aimee S. James

**Affiliations:** 1 Department of Diagnostic Medicine and Pathobiology, Kansas State University, 103 Trotter Hall, 1710 Denison Avenue, Manhattan, KS 66502 USA; 2 Applied English Center, University of Kansas, Lawrence, KS USA; 3 Metropolitan Community College Penn Valley, Kansas City, MO USA; 4 Department of Public Health Sciences, Wichita State University, Wichita, KS USA; 5 Division of Public Health Sciences, Washington University in Saint Louis School of Medicine, Saint Louis, MO USA

**Keywords:** Cultural competence, Interventions, Participatory action research, Qualitative research, Refugees, Women’s health, Family health

## Abstract

**Objective:**

To partner with and understand the health of Somali Bantu refugee women, small group sessions were designed and conducted using a community-based collaborative action research (CBCAR) approach. Health topics identified by this community were presented in 42 sessions with eleven women. Follow-up individual interviews with the women were used to ask questions about health experiences and plan for future health education. The objective of this qualitative study was to provide refugee women with knowledge to help them adjust to new health challenges in the United States, and to share personal narratives in a safe environment.

**Results:**

The process of sharing health information with the women resulted in a collaborative exchange of culture and community. Individual interviews allowed women to voice their opinions outside of the influence of their community elders. CBCAR is an effective tool to involve refugee communities, and other populations small in number, in addressing their unique health challenges. Results from this study demonstrated that small group sessions and a CBCAR approach can be effective in sharing knowledge within small communities of refugee women. Findings from the study will assist in the future planning of health education programs for refugee women and their families in this community.

## Introduction

Educational interventions for refugee families are essential for health literacy [1–3], especially due to poor health, malnutrition, and other deprivations from living in refugee camps [4–6]. Somali Bantu refugee women in the United States have experienced difficulties, including limited health literacy, trauma manifestations, decreased physical activity, and dietary changes [4–6]. These difficulties can be exacerbated by language, literacy, and cultural barriers between refugees and health care providers [7].

Engaging community members as collaborators has been effective in planning interventions with refugee communities [5, 8–10]. Community-based collaborative action research (CBCAR) was utilized to incorporate refugee community priorities into program planning and implementation [8]. Reluctance to discuss sensitive topics, including pregnancy and trauma, has been documented amongst Somali refugees [11–15]. Small group sessions in a local community center were structured to encourage open discussion, and individual interviews were conducted to share personal narratives and health knowledge of this small population [5, 11, 16, 17].

## Main text

### Methods

Health educational content was formulated through consultation and partnership with the Somali Bantu community and the refugee resettlement agency working with this community in the Kansas City metropolitan area. Forty-two small-group sessions, of approximately 90 min each, were held four times weekly for 12 months. Sessions were guided by session leaders who were trained in health education, and familiar with the cultural norms and unique needs of Somali Bantu refugee women. The study participants included eleven Somali Bantu refugee women who attended an existing refugee resettlement agency program (Table 1). The sessions and interviews were interpreted between English and Maay-maay, the native language of the Somali Bantu, with interpreters that were born in Somalia and trained by an interpreter training program. The topics were presented with facilitated discussions between one and thirteen times (Table 2). The first and second author kept extensive notes, and discussed and de-briefed each other with the interpreter after each session to ensure accuracy of content. The first and second author and interpreter conducted interviews lasting approximately 30 min with the women in their homes to better understand their health and personal narrative. The interview transcripts were hand-written by the second author, and all women were shown what was written. The interviews were not audio-taped as the women expressed reluctance in being audio-taped. To ascertain learning and retention, key learning concepts were established prior to the interviews to determine if the women recalled session information.

**Table 1:** A description of the demographics of the study population

Mean	Participants (range) n = 11
Age (years)	29.3 (20–44)
Time in US (months)	14.6 (9–23)
Time in Refugee Camps (years)	8.5 (6–15)
# children	4.4 (1–7)
# children born in refugee camps	3.5 (1–5)
# children born in US	0.3 (0–1)

**Table 2:** Outcomes of health educational sessions

Session topic^a^	# of sessions	Attendance^b^	Retention^c^	Findings
Family health	5	6.0	45.5	Interest in discussion of household hygiene
Oral hygiene	1	6.0	0	Shared personal narratives of traditional methods
Personal safety	4	6.5	45.5	Shared personal narratives of personal safety in their home country
Nutrition	13	5.8	63.6	Shared personal narratives of malnutrition
Vaccination	1	8.0	0	Lack of knowledge of vaccinations across the lifespan, other than childhood
Child safety	2	7.5	0	Unfamiliar with risks of leaving children unsupervised
Prenatal health	4	6.3	9.1	Reluctance about prenatal care
Body image/weight	3	4.6	0	Interested in discussion of health and weight
Diabetes	1	5.0	0	Lack of knowledge of non-gestational Diabetes
Obesity	2	4.5	0	Inability to select healthy from non-healthy foods
Sexually transmitted diseases	2	6.5	0	Culturally inappropriate for discussion before marriage
Sexual behavior	2	6.5	9.1	Culturally inappropriate for discussion before marriage
Mental health	2	5.5	0	Inconsistency between personal narrative and recognition of symptoms

^a^Topics listed in chronological order of first presentation

^b^The mean number of women (n = 11) attending each of the session(s) for that topic

^c^Retention by the women was measured by the total number of women that remembered the session topic, as a percentage of the total number of women. Pearson’s co-efficient (r) was used to determine correlations between two variables (the number of times a topic was presented and the number of women that retained the topic) to determine if retention of information presented during small group sessions was predictive of the frequency of topic presentation

Demographic data were collected from agency records and confirmed during interviews. The order of the interviews was random, and interviewees were randomly assigned a number to assure confidentiality. The key assigning the interview number to the participant was maintained in a separate file from all other data. All women provided informed consent verbally prior to the study. All interview procedures and data collection methods were approved by the Refugee Resettlement agency and by the Institutional Review Board of the University of Kansas Medical Center. The results from the interviews were tabulated and analyzed using Microsoft Excel and SAS 9.1. Pearson’s co-efficient (r) was used to determine correlations between two variables to determine if retention was predictive of topic frequency. The written interview transcripts and notes were reviewed by the first and second author and interpreter, and corrected to assure accuracy. The transcripts and notes were read again independently, and codes were assigned using CBCAR to guide the deductive development of codes for qualitative analysis of thematic content. To assure accurate coding of the data, the authors and the interpreter also discussed and confirmed agreement for the identified recurring patterns and emerging themes. The corrected, typed transcripts and notes were entered into NVIVO 10.0 (QRS International LTD, 2016) to classify, sort and analyze the data.

### Results

#### Participant characteristics

The refugee women spent an average of 8.5 years in refugee camps. The average age of the women was 29.3 years. The women had an average of four children each. Of the 48 total children, six were born prior to living in the refugee camps, 39 were born in the refugee camps, and three were born in the United States. The marital status of the women included married (72.7%), divorced (18.2%) and widowed (9.1%).

#### Health Education

The most frequently presented topic was nutrition based on requested interest of the women (Table 2). The women had suffered from malnutrition in refugee camps and were unfamiliar with many foods in the US. The women indicated that their diet was limited in the refugee camps, and commented:

“We were hungry a lot of the time.”

“Some days we waited a long time for food.”

“We had plenty of food when we lived on our farms.”

Nutrition was also discussed in terms of food preparation, healthy and non-healthy foods, and why certain foods are good for prenatal health. During this study, six women were pregnant, and all women indicated that they had breast-fed their children. Therefore, four sessions were dedicated to nutrition during pregnancy and breast-feeding.

Diabetes and obesity were discussed during one and two sessions, respectively. The women indicated that they thought diabetes only occurred during pregnancy. When the women were shown soda beverages, alcoholic beverages, and potato chips together in a picture and asked which items were unhealthy, they indicated only the alcohol. The women indicated that they thought healthy babies were “not skinny”. During some sessions, the women prepared ethnic dishes to share, which were observed to be high in oil, salt, and carbohydrate content. In addition, the women reported their physical activity and daily exercise were decreased.

Reproductive health, including reproduction, contraception, and childbirth, was discussed during two sessions. When asked about prenatal visits, inducing labor, and caesarean surgery, the women expressed fear and reluctance. Some comments included:

“American doctors always want to cut and look inside.”

“My husband does not like the doctor to look inside.”

Post-traumatic stress disorder (PTSD) because of living in refugee camps and trauma was concerning to the community. The three session leaders had worked with refugee populations previously, two session leaders were culturally representative, and discussed PTSD in terms of “feeling sad.” The women indicated that they did not suffer from any symptoms described. This was contrasted with their previous descriptions of hardships in refugee camps, including difficulties with health care and suffering from violent physical attacks:

“We had no doctor.”

“I was hit and beaten.”

“They came and attacked me.”

#### Health behaviors, attitudes and knowledge

During the interviews, a questionnaire was used to collected qualitative data about health behaviors and knowledge, and the women were asked about what they remembered from the sessions. Of the topics presented, nutrition was the topic most retained, and was remembered by 63.6% of the women (Table 2). Ten of the eleven women named foods important for childhood health, and seven named food items important for prenatal health. This knowledge of nutrition may reflect knowledge acquired through an alternative venue to this study. All of the women said they did not worry about food. 18.2% of the women specifically mentioned that “food stamps” allowed them to buy food for their family.

Retention of the topics was compared to the number of times the topic was presented. The topic that had the highest rate of retention, nutrition, was the topic presented the greatest number of times. Analysis using the Pearson co-efficient showed a strong positive correlation between the number of times a topic was presented and the number of women that retained the topic (r = 0.852, p < 0.01). A positive, but not significant, correlation was found between the number of women attending sessions and the number of women that retained the topic (r = 0.501, p = 0.115, N.S.).

Satisfaction with access to health care was discussed. The women communicated ease related to going to the doctor or hospital, and reported being satisfied with how health professionals behaved towards them. The possession of a medical card was specifically mentioned by four women. They indicated that transportation to the doctor was easier in the United States than in Africa. The availability of interpreters was also expressed to be adequate, with seven women indicating that an interpreter was always provided.

The coded data were analyzed according to CBCAR dimensions of partnership, dialog, pattern recognition, dialog on meaning of pattern, insight into action, and reflecting on evolving pattern.

### Discussion

This qualitative study used CBCAR to guide and provide a pathway to explore, share and understand personal health narratives [8]. The six CBCAR dimensions resulted in six translated content themes for the refugee women’s experience with the program (Table 3).

**Table 3:** Themes and study findings using a community-based collaborative action research framework

Themes	Findings	CBCAR dimension
Develop community and trust	Relationship built over the study time period was essential to gain community trust	Partnership
Provide a space to share personal opinions	Creation of a safe and private venue was crucial for open dialog	Dialog
Explore meaning of shared experiences and personal narratives	Sharing of personal opinions in safety of private interviews gave greater depth to understanding	Pattern recognition
Understand and appreciate influence of life experiences on current health	Mutual understanding of the lives of participants and team members facilitated success of the project	Dialog on meaning of pattern
Identify a path forward for continued health and community	Recognition of community concerns and knowledge gaps allowed for better planning for future sessions	Insight into action
Evaluate success and consider new understandings	Reflected on the outcomes and future directions, formed meaningful relationships with community	Reflection on evolving pattern

Partnership: the development of community and trust over the extended time period of the study was essential to building relationships. The ethnicity and gender of session leaders and interpreters was a relevant finding. During the program, it was learned that the women viewed an unmarried interpreter as unacceptable for discussions of pregnancy. To address this, a female, married interpreter was used for all the interviews to be culturally consistent and better ensure the women would feel comfortable. In addition, the primary author was viewed as marginally acceptable at the beginning of this program, although married, as she did not have any children.

Dialog and pattern recognition: the sessions attended by the women and the research team were intentionally designed to share personal stories and to have open dialog. This sense of privacy was enhanced in the individual interviews. The interviews allowed the women to express their individual opinion in private, separate to those expressed during the sessions. In sessions, the women generally did not express individual opinions, but rather expressed a community opinion. From continued interaction, it became clear that the women respected a cultural hierarchy, in which one older woman was looked to for guidance. The women who did not follow the guidance of the elder woman were not blood relatives or relatives by marriage. It is likely that the women followed the opinion of the female authority figure during the sessions, but in the individual interviews expressed their own opinions. This allowed for a greater understanding and exploration of shared experiences that both participants and research team had as women, daughters, wives and mothers.

The final stages of CBCAR illustrate the actions that transform and impact community health [8].

Dialog on meaning of pattern: to understand the influences of life experiences on health, shared discussions allowed for a deeper mutual appreciation of all participants and authors for each other’s lives, which facilitated success of this project.

Insight into action: to identify a path forward, recognition of community concerns and knowledge gaps will allow for better planning for future sessions with this community.

Reflection on evolving pattern: to evaluate programmatic success, and consider new understandings within the community, the research team and women reflected on the outcomes and future directions. The formation of a meaningful relationship was communicated by the participant community as the most important study outcome and success.

### Conclusions

The results of this qualitative study hold promise for further research and implications of future educational programs and action planning with these Somali Bantu refugee women [5, 11]. To apply the findings of this study to future collaborations with this community, facilitators who engage the community, and are sensitive to cultural norms, are essential to instill trust and confidence needed for honest discussion, as well as to effectively address topics that participants may be reluctant to discuss. Facilitators should also be aware to interview participants individually to ensure that an individual opinion is shared, in addition to focus groups where a group opinion may be offered.

## Limitations

The small sample size and non-randomized design of this qualitative study limit the generalizability of the findings for other refugee populations. The authors also recognize the limited external validity of this study, and the need for future studies with a larger sample size. Partnering with populations small in number is challenging, however, it is important to better understand the health knowledge and attitudes of this and other small populations [16, 17]. Future partnership and stakeholder co-operation focused on community priorities are vital to successfully tackle significant health challenges identified by this community.

## Data Availability

Identifying and/or confidential participant data cannot be shared. However, data generated or analyzed during the current study are available from the corresponding author on reasonable request.

## Abbreviations

CBCAR: community-based collaborative action research
PTSD: post-traumatic stress disorder
